# Protein Kinases and Neurodegenerative Diseases

**DOI:** 10.3390/ijms24065574

**Published:** 2023-03-14

**Authors:** Ichiro Kawahata, Kohji Fukunaga

**Affiliations:** 1 Graduate School of Pharmaceutical Sciences, Tohoku University, Sendai 980-8578, Japan; 2 BRI Pharma Inc., Sendai 982-0804, Japan

Global aging has led to an increase in age-related neurological disorders, which have become a societal problem. Understanding the regulation of neuronal homeostasis is essential for unveiling the pathogenic mechanisms of intractable neurodegenerative diseases, leading to therapeutic applications and the discovery of novel drug targets. This Special Issue, which contains three original research papers and two reviews written by a panel of experts who highlight recent advances in protein kinases and their relationship with neurodegenerative diseases, aims to advance our understanding of the unique regulation of neuronal homeostasis and elucidate the mechanism of pathogenesis mediated by protein kinases in human neurodegenerative diseases. It also explores individual therapeutic targets and drugs and demonstrates their efficacy and mechanisms to challenge the preparation of unique therapeutics.

Various protein kinases, including those in the keywords of this Special Issue, regulate cellular homeostasis. The phosphoinositide 3-kinase (PI3K), AKT (also known as protein kinase B), and mammalian target of rapamycin (mTOR) signaling pathways regulate various cellular processes such as growth, proliferation, survival, and metabolism. PI3K converts phosphatidylinositol 4,5-bisphosphate (PIP2) to phosphatidylinositol 3,4,5-trisphosphate (PIP3), which then recruits AKT to the cell membrane. Once activated, AKT phosphorylates and regulates downstream targets such as mTOR. Dysregulation of the PI3K/AKT/mTOR pathway has been implicated in many diseases including cancer, diabetes, and neurological disorders. Therefore, this pathway is an important target for drug development and therapeutic interventions. Another kinase, AMP-activated kinase (AMPK), is an enzyme that acts as a central regulator of metabolism in mammalian cells. AMPK promotes energy-producing catabolic processes such as glucose uptake and fatty acid oxidation, while inhibiting energy-consuming anabolic processes such as protein and lipid synthesis. Consequently, the dysregulation of AMPK has been implicated in several diseases, including type 2 diabetes, obesity, and cancer. Therefore, AMPK has become a target for drug development to help treat these diseases, and its activation is associated with potential health benefits.

Apoptotic signal-regulating kinase 1 (ASK-1) and p38 mitogen-activated protein kinase (p38 MAPK) are involved in cellular responses to stresses, including oxidative stress, endoplasmic reticulum (ER) stress, and cytokine signaling. The ASK-1/p38 pathway is initiated by various stress signals, such as reactive oxygen species, which leads to the activation of ASK-1. ASK-1 then activates p38 MAPK via phosphorylation. Once activated, p38 MAPK phosphorylates downstream targets, such as transcription factors, kinases, and other signaling proteins, leading to the regulation of various cellular processes, such as inflammation, apoptosis, and cell differentiation. The ASK-1/p38 signaling pathway has been implicated in several diseases, including cancer, cardiovascular and neurodegenerative diseases, and immune disorders. Calcium/calmodulin-dependent protein kinase II (CaMKII), which is activated by calcium and calmodulin, is a key signaling molecule in several cellular processes, including learning and memory, synaptic plasticity, and gene expression. Activated CaMKII can phosphorylate various downstream targets, such as ion channels, transcription factors, and synaptic proteins, leading to changes in cellular function and gene expression. In the brain, CaMKII is critical for synaptic plasticity and plays a role in long-term potentiation (LTP). Consequently, the dysregulation of CaMKII has been implicated in several diseases, including Alzheimer’s disease, schizophrenia, and cardiovascular diseases. Therefore, CaMKII is a potential target for the development of drugs for the treatment of these diseases.

This Special Issue has led to several exciting discoveries regarding the development of therapeutic drugs targeting protein kinases. Ginsenoside Re (GRe) is a natural compound found in the roots of *Panax ginseng*, which is widely used in traditional Eastern medicine. GRe is a saponin with a wide range of pharmacological effects, including anti-inflammatory, anticancer, antioxidant, antidiabetic, and neuroprotective properties. Therefore, the potential to improve cognitive function is expected. In this context, Shin et al. clearly demonstrated that protein kinase Cδ (PKCδ) is a therapeutic target for GRe against serotonergic impairment by inhibiting mitochondrial translocation, thereby reducing mitochondrial glutathione peroxidase activity, mitochondrial dysfunction, and oxidative stress [1]. PKCδ, which belongs to a group of serine/threonine kinases, is involved in the regulation of various signaling pathways, such as the NF-κB, JNK pathway, and MAPK/ERK pathways, and is widely expressed in various tissues and is involved in various biological functions, including cell survival, proliferation, and differentiation. Therefore, PKCδ has been implicated in several pathophysiological conditions such as cancer, cardiovascular diseases, and neurodegenerative diseases. Thus, elucidating the PKCδ-targeting mechanism in this study is promising for the development of therapeutic drugs for neuronal dysfunction.

Another study focused on the degradation of pathogenic proteins. Xu et al. demonstrated that CaMKII activation led to proteasome activation and ameliorated α-synuclein pathology in a mouse model of dementia with Lewy bodies (DLB) [2]. DLB is a neurodegenerative disease characterized by the presence of abnormal protein deposits, called Lewy bodies, in the brain. These Lewy bodies are defined by aggregates of a pathogenic protein called α-synuclein. The researchers discovered that a novel small molecule, SAK3, a T-type calcium channel enhancer, activated CaMKII and Rpt-6 to increase proteasomal activity. Rpt-6, also known as 26S proteasome regulatory subunit 8 (PSMC5), is a component of the 26S proteasome responsible for degrading misfolded proteins in the cell. As a result, SAK3 successfully reduced α-synuclein deposition in DLB pathology in the mouse model, followed by the recovery of memory and cognition. These data also suggest the therapeutic potential of SAK3 for another α-synucleinopathy, Parkinson’s disease. These findings will accelerate the development of therapeutic drugs for Lewy body diseases.

This Special Issue also reports the physiological significance of c-Jun N-terminal kinase 3 (JNK3) and its potential therapeutic applications in cultured cell models. JNK3 is a member of the mitogen-activated protein kinase (MAPK) family of enzymes that is primarily expressed in the brain and is involved in various pathological processes, including neuronal development, synaptic plasticity, and neuronal apoptosis. Dysregulation of JNK3 activity is linked to oxidative stress, mitochondrial dysfunction, and neuroinflammation in neurological disorders, including Alzheimer’s, Parkinson’s, and Huntington’s diseases. In this context, Rehfeldt et al. evaluated the neuroprotective, anti-inflammatory, and antioxidant effects of a synthetic compound (FMU200) with known JNK3 inhibitory activity in SH-SY5Y and RAW264.7 cell lines [3]. FMU200 successfully reduced the number of both early and late apoptotic cells, decreased ROS levels, restored mitochondrial membrane potential, and downregulated JNK phosphorylation and tumor necrosis factor-α (TNF-α) levels. In Alzheimer’s disease, chronic neuroinflammation, including the activation of microglia and astrocytes and the production of proinflammatory cytokines such as TNF-α, contributes to neuronal damage and cognitive decline. In addition, TNF-α participates in the inflammatory process by promoting the production of reactive oxygen species and impairing mitochondrial function in Parkinson’s disease. The results of this study suggest that FMU200 is a potential therapeutic candidate for treating neurodegenerative diseases.

The accumulation of α-synuclein in the brain is strongly implicated in the pathogenesis of DLB and Parkinson’s disease. However, it is not well understood why proteins accumulate and aggregate in the brain and how kinases are involved. Lewy bodies, which are present in the neurons of patients with sporadic Parkinson’s disease, DLB, and glial cytoplasmic inclusion bodies in oligodendrocytes with multiple system atrophy, are mostly composed of highly phosphorylated and improperly aggregated α-synuclein. Cytotoxic aggregated α-synuclein is a major factor involved in the development of synucleinopathies. Most α-synuclein in a healthy brain is unphosphorylated; however, more than 90% of the abnormally aggregated α-synuclein in the Lewy bodies of Parkinson’s disease patients is phosphorylated at Ser129. Ser129 is phosphorylated by a number of kinases, although the function of phosphorylating enzymes in disease pathogenesis and their relationship with the cellular toxicity of phosphorylation in α-synucleinopathy are still poorly understood. In this context, our review with Prof. Finkelstein focused on G protein-coupled receptor kinase (GRK), casein kinase II (CKII), and polo-like kinase (PLK) and their physiological significance on α-synuclein phosphorylation [4]. We also summarize the therapeutic applications of inhibitors of the abovementioned kinases and their pharmacological effects in cell culture and animal models. Accordingly, the phosphorylation of α-synuclein and its accumulation with disease progression is a potential therapeutic target and diagnostic biomarker.

Protein kinases are also associated with the pathogenesis of other intractable diseases, including amyotrophic lateral sclerosis (ALS) and frontotemporal dementia (FTD). Therefore, this Special Issue focuses on immune-related signaling kinases associated with these two diseases. ALS, also known as Lou Gehrig’s disease, is a progressive neurodegenerative disorder that affects neuronal cells in the brain and spinal cord, leading to muscle weakness, stiffness, and eventually paralysis. Because FTD affects the frontal and temporal lobes, which are essential for language, behavior, and decision making, it can cause significant changes in a person’s personality, behavior, and language skills. In this context, Garcia et al. summarized the related kinase cascades, including TANK-binding kinase 1, receptor-interacting kinase 1, receptor of activated protein C kinase 1, AMP-activated protein kinase, and leucine-rich repeat kinase 2, and their importance in the pathogenesis of ALS and FTD [5]. They also introduced potential therapeutic inhibitors of the abovementioned kinases, including masitinib, a tyrosine kinase inhibitor used to treat various types of cancer and inflammatory diseases, and fasudil, a calcium channel blocker and Rho-kinase inhibitor used to treat various diseases, including stroke, heart failure, and Alzheimer’s disease. This review highlights the fact that considerable work remains to be conducted to elucidate the complex mechanisms underlying the pathogeneses of ALS and FTD, which will lead to the identification of novel kinase therapeutic targets.

These original papers and review articles demonstrate significant progress in elucidating the molecular mechanisms of intractable diseases and their therapeutic applications. Furthermore, these studies have identified areas where there are missing links in kinases that need to be clarified and resolved during the loss of neuronal homeostasis. The authors also suggest that existing inhibitors may be effective in treating neurodegenerative diseases. For example, masitinib has been approved in Europe for the treatment of mast cell tumors in dogs and is studied in humans for inflammatory diseases, such as multiple sclerosis, rheumatoid arthritis, and asthma. The elucidation of novel disease mechanisms will lead to the discovery of drug targets and unique compounds. In addition, the unveiled kinase cascades in pathogenesis will lead to breakthroughs in the expansion of indications for existing drugs and drug repositioning. These achievements will help us overcome intractable neurological diseases and realize a society with brain health and longevity.
